# FiberWire tension band for patellar fractures

**DOI:** 10.1007/s10195-015-0359-6

**Published:** 2015-07-05

**Authors:** Lawrence Camarda, Alessandra La Gattuta, Marcello Butera, Francesco Siragusa, Michele D’Arienzo

**Affiliations:** Department of Orthopaedic Surgery, DICHIRONS, University of Palermo, Via del Vespro, 90100 Palermo, Italy

**Keywords:** Patellar fracture, Knee fracture, FiberWire, Tension band, Suture

## Abstract

**Background:**

Symptomatic hardware represents the most frequent complication reported following surgical treatment of patellar fracture. For this reason, some authors suggested using nonabsorbable sutures to fix the fracture with various techniques. The aim of this study was to evaluate clinical and radiological results of patients treated following a modified Pyriford technique using a FiberWire suture (Arthrex, Naples, FL, USA).

**Materials and methods:**

We retrospectively evaluated a case series of  seventeen patients with displaced patellar fractures treated by open reduction and internal fixation with a modified tension band using FiberWire sutures. Clinical and radiological outcome were evaluated. Union time, complications, and reoperation rate were observed and recorded.

**Results:**

All fractures healed (time to union 9.2 ± 2 weeks), and no fixation failure was observed. Slight losses of reduction (<4 mm) were noted in two patients at 4 weeks postoperatively. The average Lysholm and Bostman scores at the final follow-up were 91 ± 5.7 (range 83–100) and 28.3 ± 1.6 (range 26–30), respectively.

**Conclusion:**

Modified tension band using FiberWire sutures showed satisfactory clinical results, with a low incidence of complications and reoperations. FiberWire tension bands could be used in place of metal-wire tension bands to treat patellar fracture, reducing the rate of symptomatic hardware.

**Level of evidence:**

4

## Introduction

Patellar fractures are relatively uncommon, representing approximately 1% of all skeletal injuries [2]. Nonsurgical management is recommended for fractures with intact extensor mechanism, minimal intra-articular stepoff, and minimal fracture displacement (1–4 mm) [3]. An incompetent extensor mechanism associated with a displaced or comminuted fracture with a torn extensor retinaculum is indicated for surgery. Operative treatment must provide stable patellar fracture fixation to allow early mobilization and prevent fracture displacement. Furthermore, articular congruity is essential to reduce the increased risk of posttraumatic osteoarthritis as a result of the high-contact forces in the patellofemoral joint [5].

Several different techniques for internal fixation have been proposed and employed over the years, with different rates of success. Historically, the most commonly used technique for managing patellar fracture fixation is represented by the modified tension-band wiring technique [5, 13, 19, 23]. It involves longitudinal Kirschner wires (K-wires) and 18-gauge stainless steel wire in a figure-of-eight pattern looped over the anterior side of the patella. This technique neutralizes tension forces anteriorly produced by the extensor mechanism at knee flexion and converts them into stabilizing compressive forces at the articular surface [23]. This construct represents the most widely used method of fixation for transverse and comminuted patellar fracture. Over the years, this technique was further modified by different authors using either K-wires or cannulated screws with different stainless steel wire configurations. Even though substantially good clinical results have been reported, all techniques using metallic wires can result in symptomatic hardware, which represents the most frequent complication, with reported rates varying from 0 % to 60 % [12, 14, 20]. In fact, implants can irritate overlying soft tissue and cause pain, requiring a second surgery to remove the implant. In addition, a high incidence of infection has been reported [9, 12]. For this reason some authors have advocated the use of nonabsorbable sutures, such as braided polyester, to fix the fracture when using various techniques [6, 7, 9, 12, 21]. Several advantages of using a suture over a wire have been shown in the literature, such as lower rate of revision surgery and higher patient tolerance of surgical material that does not irritate soft tissues as much as wire. Another advantage is represented by easier handling of suture materials for the surgeon, which could determine shorter operating and tourniquet time [9, 11]. Few authors reported data concerning the use of tension-band fixations employing nonabsorbable sutures. Although no. 5 Ethibond (Ethicon, Somerville, NJ, USA) and no. 5 Ti-Cron (Davis and Geck, Gosport, Hampshire, UK) braided polyester sutures have been clinically and biomechanically studied for patellar fracture fixation, to our knowledge there are no data on the use of FiberWire sutures (Arthrex, Naples, FL, USA).

The aim of this study was to evaluate clinical outcome and rate of postoperative complications of patients who underwent an open reduction and internal fixation (ORIF) using a tension-band wiring technique with a FiberWire suture. The authors hypothesized that a FiberWire tension band could be used for patellar fracture fixation, decreasing the rate of symptomatic hardware without reducing the rate of fracture healing.

## Materials and methods

We performed a retrospective study of consecutive patients hospitalized for patellar fractures in our Institution (Orthopaedic and Traumatology Unit, AOUP “P.Giaccone”, Palermo, Italy) from 2008 to 2013. Inclusion criteria for the study were the use of FiberWire suture for fracture fixation, age ≥18 years, and minimum follow-up of at least 10 months. Exclusion criteria were open fractures, stainless steel wire implantation, previous ipsilateral knee surgery, and patients with polytrauma or head injuries that definitely influence rehabilitation.

Fifty-one patients with patellar fractures were admitted to the hospital during the mentioned period. Among them, 37 required ORIF. Seventeen patients were excluded: specifically, eight were treated with a suture other than the FiberWire; in six patients, a hybrid technique was used (metallic K-wires and nonabsorbable suture); two patients presented and open fracture; one patient presented with polytrauma that required long hospitalization, compromising the rehabilitation program. Twenty patients met inclusion criteria. All patients were contacted by telephone and/or email and were asked to return to the hospital for a final physical examination. In order to maximize follow-up, Internet-based searches was used to contact patients who did not respond to the first mailing. Three patients were lost to follow-up: one patient refused the final clinical assessment because she was living abroad; two patients could not be located. For all patients, medical records and X-ray films were reviewed and fracture types classified according to the Orthopaedic Trauma Association fracture classification (AO/OTA) system.

**Fig. 1:**
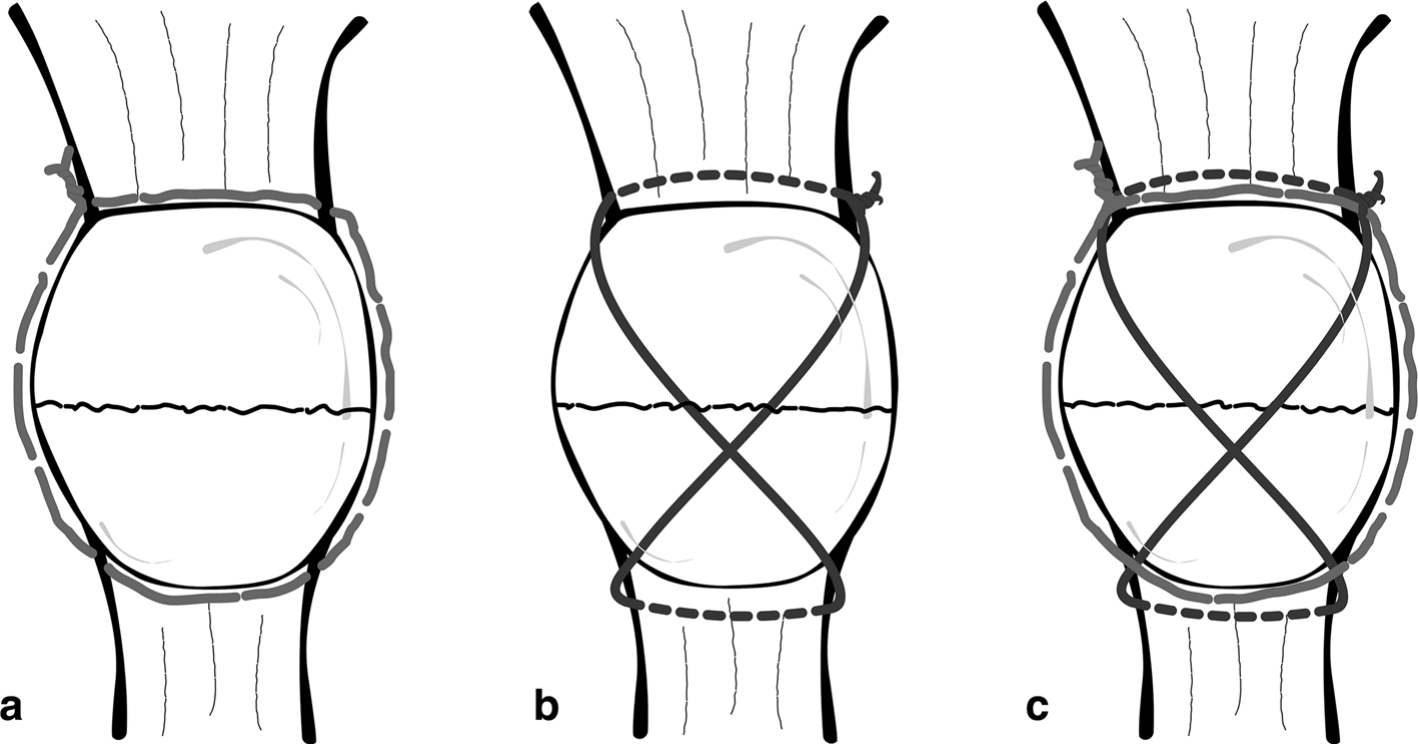
FiberWire tension band. Peripatellar circumferential cerclage (**a**); modified anterior tension band (**b**); final construct (**c**)

In all patients, surgery was scheduled 2–3 days after tje trauma and was performed by the senior author. Patients were operated on in a supine position, and a tourniquet was placed high up on the thigh. In all cases, a longitudinal skin incision on the knee was performed. An extemporary reduction was performed with a reduction clamp and checked under fluoroscopy. In this phase, care was taken to obtain the best possible reduction and congruence of the articular patellar surface. At this point, a modified Pyriford technique was used for fracture fixation [8]. A no. 5 FiberWire nonabsorbable suture was used, and a peripatellar circumferential cerclage was performed in a purse-string fashion close to the bone. This allowed initial fracture stabilization. A second no. 5 FiberWire suture was then placed in a figure-of-eight fashion through the quadriceps and patellar tendon to obtain a modified anterior tension band. Each of the two FiberWire bands was manually tensioned and knotted on the superior board of the patella (Fig. 1). Additionally, retinacular defect was repaired with an absorbable suture.

All patients were immobilized postoperatively with a long-hinged knee brace locked at 0° for 3 weeks. Weight bearing started as tolerated with crutches, while full weight bearing was allowed from the fourth week postoperatively. Passive range of motion (ROM) began at the third week postoperatively and was limited at 0–90° for the next 2 weeks, allowing active flexion/extension; full ROM was allowed from the sixth week postoperatively.

Anteroposterior and lateral radiographs of the knee were obtained every 2–4 weeks until achievement of bony union and again at final follow-up. Details of mechanism of injury, associated injuries, timing of surgery, time to fracture healing, postoperative complications, final knee ROM, and implant removal were recorded. Time requested to regain normal daily activity was also scheduled. In addition, knee function was evaluated according to Bostman and Lysholm/Tegner scores [1].

## Results

Of 20 patients, 17 were available for clinical and radiological follow-up (12 men and 5 women). The main mechanisms of injury were fall from height on hard ground in ten cases (59 %) and road traffic accidents in the remaining seven (41 %). Average patient age at injury was 46.6 years (range 20–75). There were nine transverse fractures, six comminuted, and two of the inferior pole. The average follow-up was 33 months (range 8–68). No patient had significant flexion contracture. Average ROM was 131.1° for flexion (range 120°–140°) and 0.5° for extension (range 0°–3°). No significant ROM differences were noted with the uninjured contralateral knee. Average Bostman scores at 3 months postoperatively and final follow-up were 25.2 ± 2 (range 20–30) and 28.3 ± 1.6 (range 26–30), respectively. Further, the mean Lysholm score at final follow-up was 91 points ±5.7 (range 83–100).

**Fig. 2:**
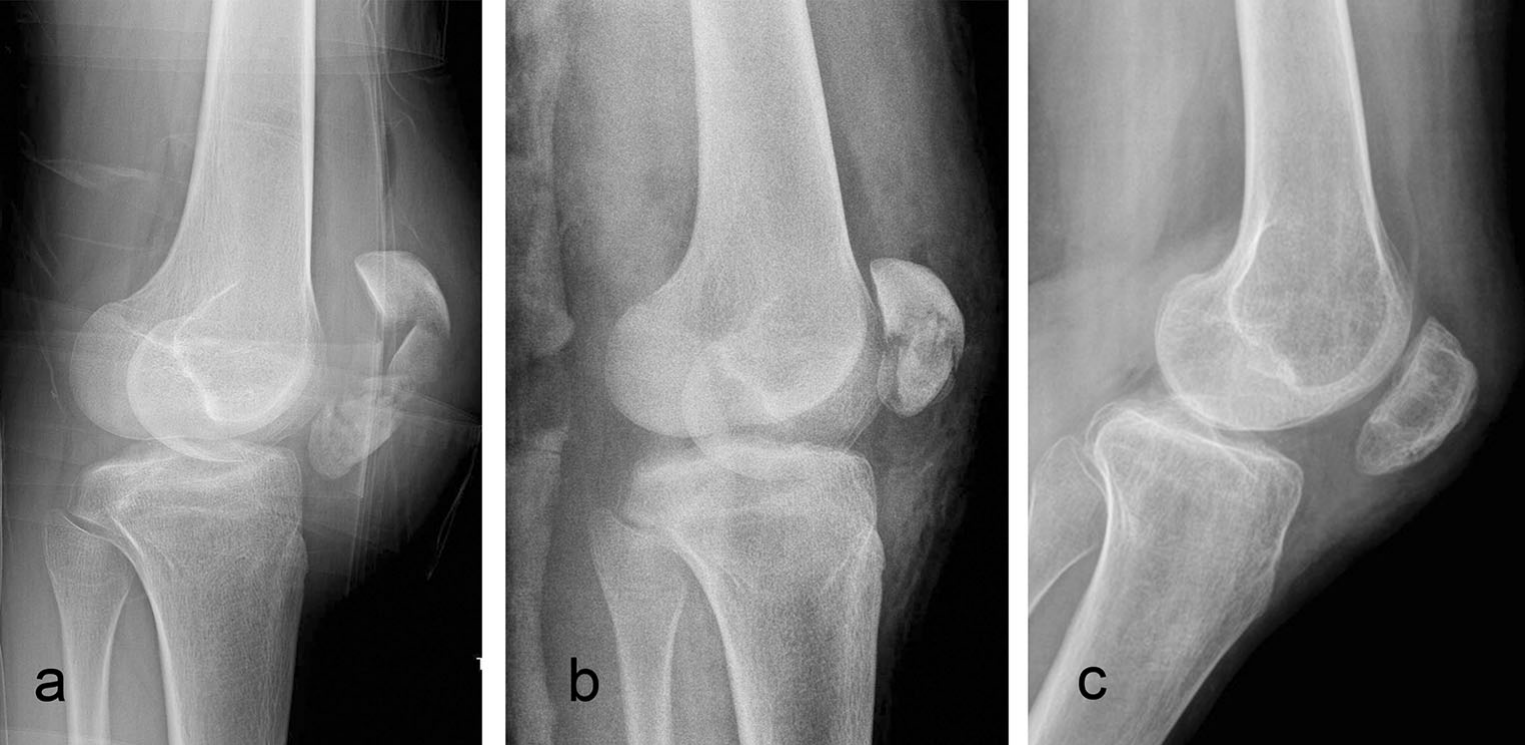
Comminuted patellar fracture. Preoperative X-ray (**a**); postoperative X-ray (**b**); final follow-up X-ray (**c**)

**Fig. 3:**
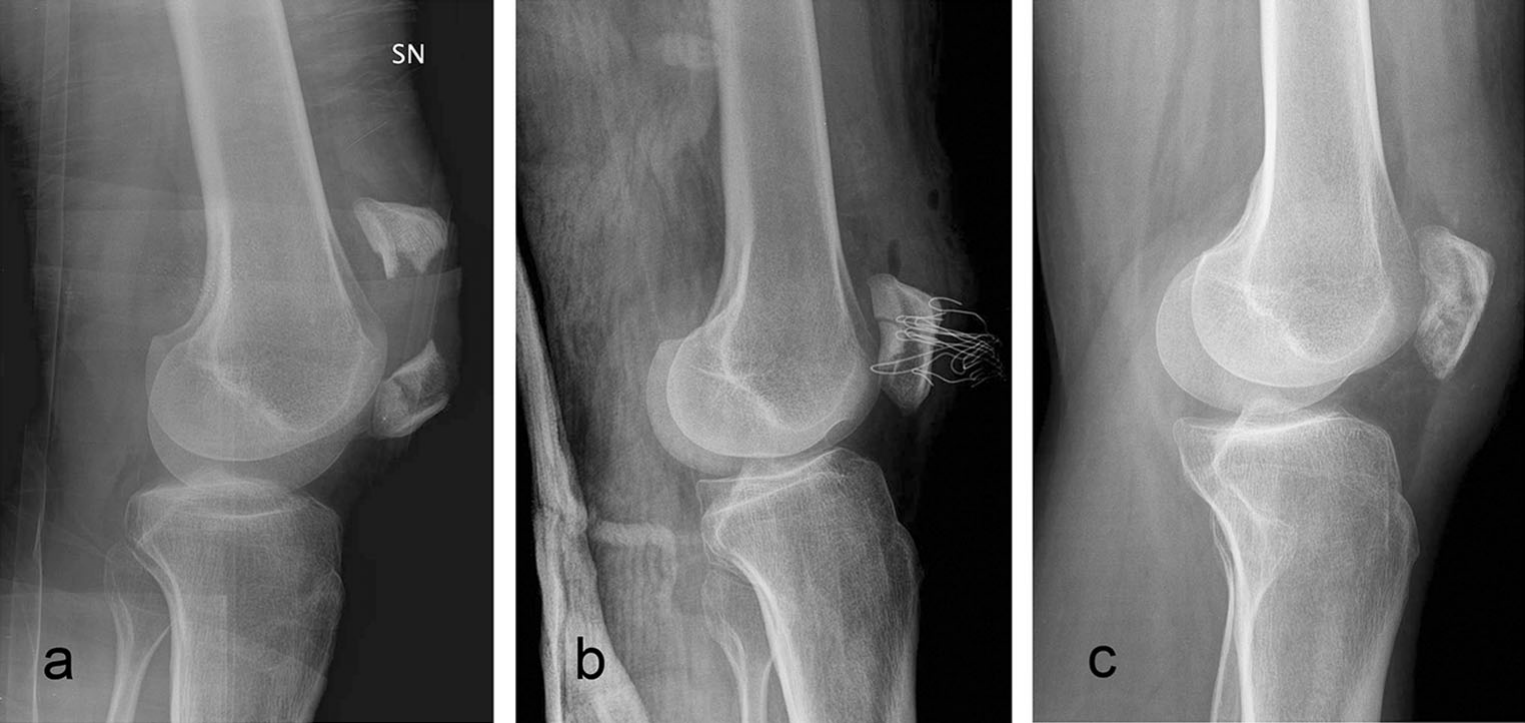
Transverse patellar fracture. Preoperative X-ray (**a**); postoperative X-ray (**b**); final follow-up X-ray (**c**)

All fractures healed (time to union 9.2 ± 2 weeks) and no fixation failure was observed in the group studied. Slight losses of reduction (<4 mm) were noted in two patients at 4 weeks postoperatively [19, 22]. Because of noncompliance with the postoperative rehabilitation protocol, one patient presented knee stiffness at 2 months postoperatively that required gentle manipulation under anesthesia (Fig. 2). The same patient was the only one in the study to requrie elective FiberWire removal 24 months after surgery. This was performed secondary to a superficial infection, which did not affect the final clinical outcome (ROM 0–125°). Another patient underwent knee arthroscopy in another Institution because of anterior knee pain. No patients referred localized pain deriving from prominent suture knots (Fig. 3). Demographics, fracture type, and outcomes are presented in Table 1.

**Table 1:** Demographic data, clinical outcomes, and postoperative complications of patients who underwent open reduction and internal fixation using a tension-band-wiring technique with a FiberWire suture

Patient	Age (years)	Type of patellar fracture	Follow-up (months)	Bostman scores	Lysholm scores	Complication
1	40	Transverse	68	26	83	–
2	36	Transverse	66	30	90	–
3	43	Inferior pole	52	30	99	–
4	64	Comminuted	51	27	89	Slight losses of reduction (<4 mm)
5	49	Comminuted	36	26	85	Slight losses of reduction (<4 mm)
6	44	Comminuted	35	30	95	–
7	71	Comminuted	22	30	100	–
8	30	Transverse	37	30	100	–
9	22	Comminuted	24	26	83	Knee stiffness + FiberWire removal
10	51	Inferior pole	8	30	90	–
11	60	Transverse	9	28	86	–
12	75	Comminuted	12	26	85	–
13	48	Transverse	10	28	94	–
14	20	Transverse	40	28	89	Anterior knee pain
15	40	Transverse	20	27	90	–
16	65	Transverse	32	30	99	–
17	33	Transverse	43	29	95	–

## Discussion

The most important finding of our study was that FiberWire tension-band technique showed positive clinical outcomes with low implant-related complication rates. Just one of 17 patients required elective implant removal. No other patient complained of symptomatic hardware. Further, all fractures healed, and no fixation failure was noted.

Different methods have been described for treating patellar fractures. Traditionally, transverse fractures are best treated with a tension-band technique using two axial K-wires, with a figure-of-eight wire placed anteriorly [18]. This technique could be combined with the use of screws in case of more comminuted fracture types [5]. However, stainless steel wire is a difficult material to manipulate through tissues and may result in poor fracture fixation [6]. Further, high rates of metal-implant-related complications have been reported, such as K-wire migrationand skin irritation due to prominent wire knots, a very common complication, ranging from 0 % to 60 % [4, 17], possibly necessitating metal implant removal. In a case series of 27 patients, LeBrun et al. reported a hardware removal rate of approximately 52% at a mean of 6.5 years of follow-up [15]. Recently, Hoshino et al. performed a retrospective study of surgically treated patellar fractures. In this study, elective implant removal was performed in 37 % and 23 % of patients treated respectively with K-wires and cannulated screws [10].

On the basis of these results, some authors have advocated the use of nonmetallic implants, such as nonabsorbable suture and biodegradable cannulated screws [6, 7, 9, 11, 21, 25]. Many of these studies reported good outcomes for patellar fractures, with very low rate of postoperative complications. However, the concept of replacing stainless steel wire with sutures remains controversial in the literature, mainly due to uncertainty regarding rigid fracture fixation. For this reason, some authors evaluated the biomechanical properties of sutures used for patellar fractures. Chatakondu et al. found a significantly lower tensile strength for Ti-Cron sutures compared with stainless steel wire (14.80 vs 34.91 kg) [6]. McGreal et al. reported that braided polyester suture is an acceptable alternative to wire in tension-band fixation after testing cadaveric patellae for >20,000 cycles of knee flexion and extension [16]. Also, Patel et al., comparing different techniques including polyester sutures (no. 5 Ethibond), concluded that braided polyester sutures were comparable to stainless-steel wire for transverse patellar in terms of quality of fixation [19]. The FiberWire represents a particular suture characterized by a core of several small, individual strands of ultra-high-molecular-weight polyethylene covered with braided polyester suture material. It is used in a variety of orthopedic procedures, such as quadriceps/patellar tendon repairs, ACL reconstruction, rotator cuff repairs, and Achilles tendon repairs. FiberWire was evaluated biomechanically for tension-band fixation of a transverse patellar fracture: Using a three-point-bend model, Wright et al. observed that a double-strand FiberWire presented a significantly higher failure load than stainless steel wire. Furthermore, it was observed that, unlike stainless steel, FiberWire maintained its initial stiffness until failure [24]. This was confirmed by our study: Using a no. 5 FiberWire tension band, we found no significant fracture displacement following knee mobilization. Only two patients presented slight losses of reduction (<4 mm). This could be justified by progressive adhesion and adjustment of the suture through the peripatellar tissue that could be present during simple load, such as quadriceps muscle contraction. Further, high losses of reduction (>4 mm) and synthesis failure, which are potentially linked to FiberWire failure or breakage, were not noted in our case series. In addition, all treated fractures healed at 3 months postoperatively. The only patient requiring FiberWire removal was a 22-year-old man who was noncompliant with the postoperative rehabilitation protocol. In fact, this patient required gentle manipulation under anesthesia because of knee stiffness at 2 months postoperatively. Further, the same patient underwent elective FiberWire removal secondary to a superficial infection, probably due to implant-related soft-tissue irritation.

On the basis of the results of this study, we found that FiberWire tension bands can be used in place of metal-wire tension bands to treat patellar fracture, reducing the rate of symptomatic hardware.

Our study has several weaknesses. Major limitations are its retrospective nature and small size. However, our group was similar in size to other studies reporting results of patellar fractures. Further, we believe that high-quality prospective, randomized studies are required to define the effectiveness of nonabsorbable suture for internal fixation of patellar fracture.

In conclusion, the study demonstrates that a modified tension band using FiberWire sutures showed satisfactory clinical results with low incidence of complications and reoperations. Thus, FiberWire tension bands could be considered an alternative solution for treating patellar fracture, thus reducing the rate of symptomatic hardware.
